# Supplementary material for: Violation of emergent rotational symmetry in the hexagonal Kagome superconductor CsV3Sb5
Source: Nat Commun. 2024 Apr 11;15:2888. doi: 10.1038/s41467-024-47043-8 (PMC11009250; doi:10.1038/s41467-024-47043-8)
Supplement: Supplementary file 1 — Supplementary Information [file 41467_2024_47043_MOESM1_ESM.pdf]

Supplementary Information for
"Violation of Emergent Rotational Symmetry in the Hexagonal Kagome Superconductor CsV_3Sb_5"

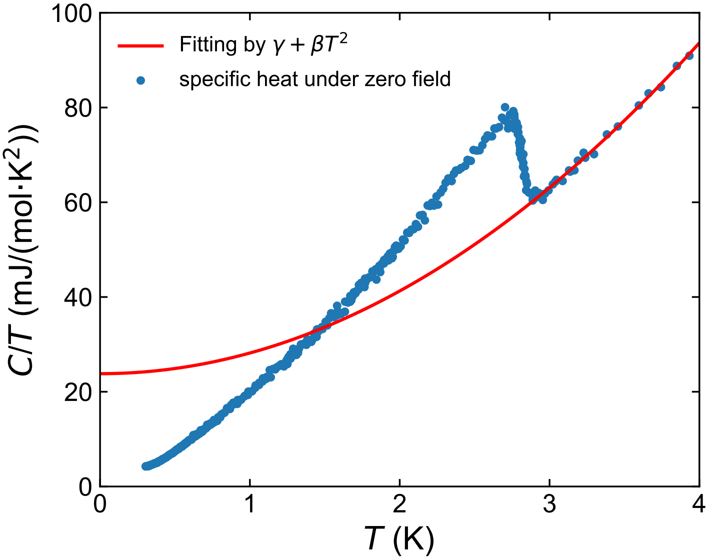

Supplementary Fig. 1 Temperature dependence of the specific heat under zero field. Background (addenda) contribution is removed. The red curve is the result of fitting of the function $C/T = \gamma + \beta T^2$ to the normal-state data from 3.1 to 4.0 K. This fitting yields $\gamma = 23.81$ mJ/K^{2}mol and $\beta = 4.364$ mJ/K^{4}mol, respectively.

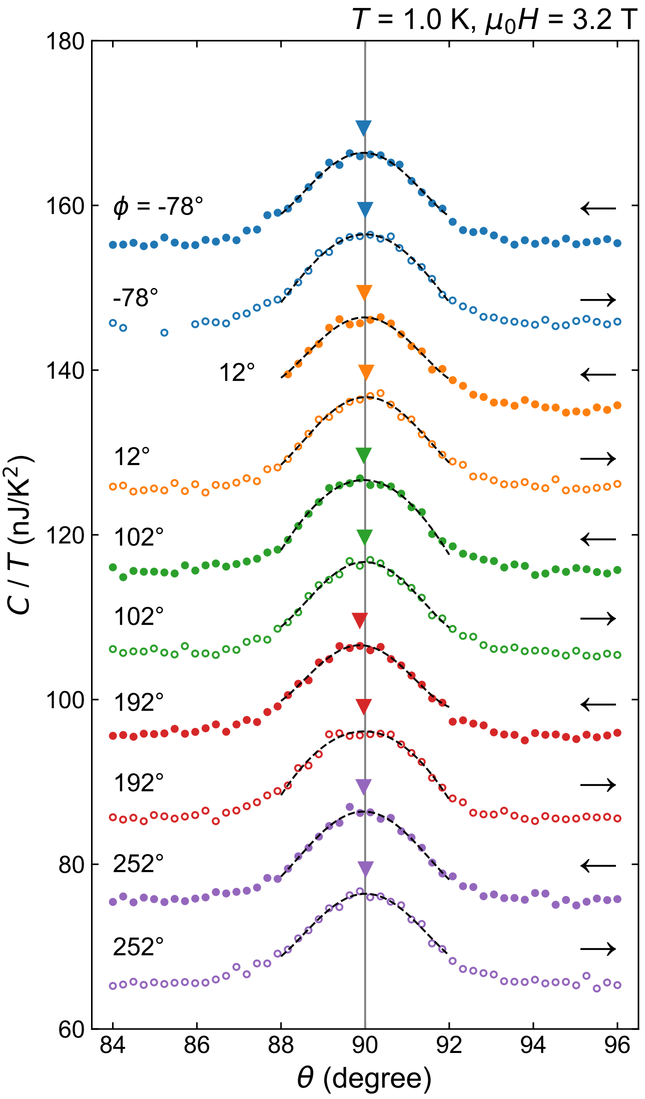

Supplementary Fig. 2 Polar-angle dependence of specific heat at several azimuth angles. Each curve is offset for clarity. The arrows means the direction of the measurement sweeps. In these plots, the specific-heat peak appears when the field is exactly parallel to the ab plane. After the conversion matrix between the laboratory and sample frames are determined, this data were measured in order to check the accuracy of the determined coordinate conversion. As one can see, for all the curves, the peak appears at $\theta = 90^\circ$ indicated by the black vertical line. Moreover, all $C(\theta)/T$ curves are symmetric with respect to $\theta = 90^\circ$. These facts manifest that the determined sample frame exactly matches with the actual sample crystalline axes. Thus, in the data presented in this paper, field misalignment is negligibly small. For quantitative analysis, we fit the peak with the even-order polynomial $f(\theta) = A_0 + A_2(\theta - \theta_{\mathrm{peak}})^2 + A_4(\theta - \theta_{\mathrm{peak}})^4$ and evaluate the peak angle θ_{peak}, which is shown with the triangles in this figure and plotted as a function of ϕ in Supplementary Fig. 3. Results of the fits are shown with the black curves. Notice that this determination of θ_{peak} makes use of not only the peak top but also the symmetry of the peak. Fitted θ_{peak} has errors of typically as small as ± 0.02 degrees, as shown in Supplementary Fig. 3.

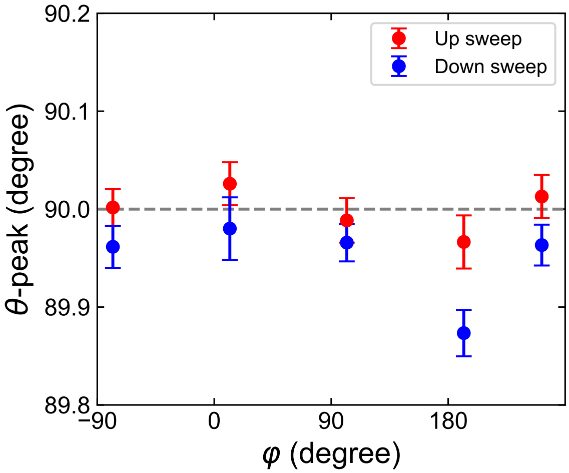

Supplementary Fig. 3 Accuracy of the magnetic-field alignment. Here, the location of the peak of $C(\theta)/T$ curves in Supplementary Fig. 2 is plotted as a function of the azimuth angle. The peak positions of the $C(\theta)/T$ curves, θ_{peak}, are obtained by fitting each data set in Supplementary Fig. 2 with a fourth-order polynomial $f(\theta) = A_0 + A_2(\theta - \theta_{\mathrm{peak}})^2 + A_4(\theta - \theta_{\mathrm{peak}})^4$. The error bar indicates the asymptotic standard error in each fitting. The red and blue symbols are obtained from up and down sweeps, respectively. The horizontal dotted line marks $\theta = 90^\circ$. Fitting this data with a sin curve gives the amplitude of about $0.04 \pm 0.02^\circ$, which provides the upper limit of the alignment accuracy.

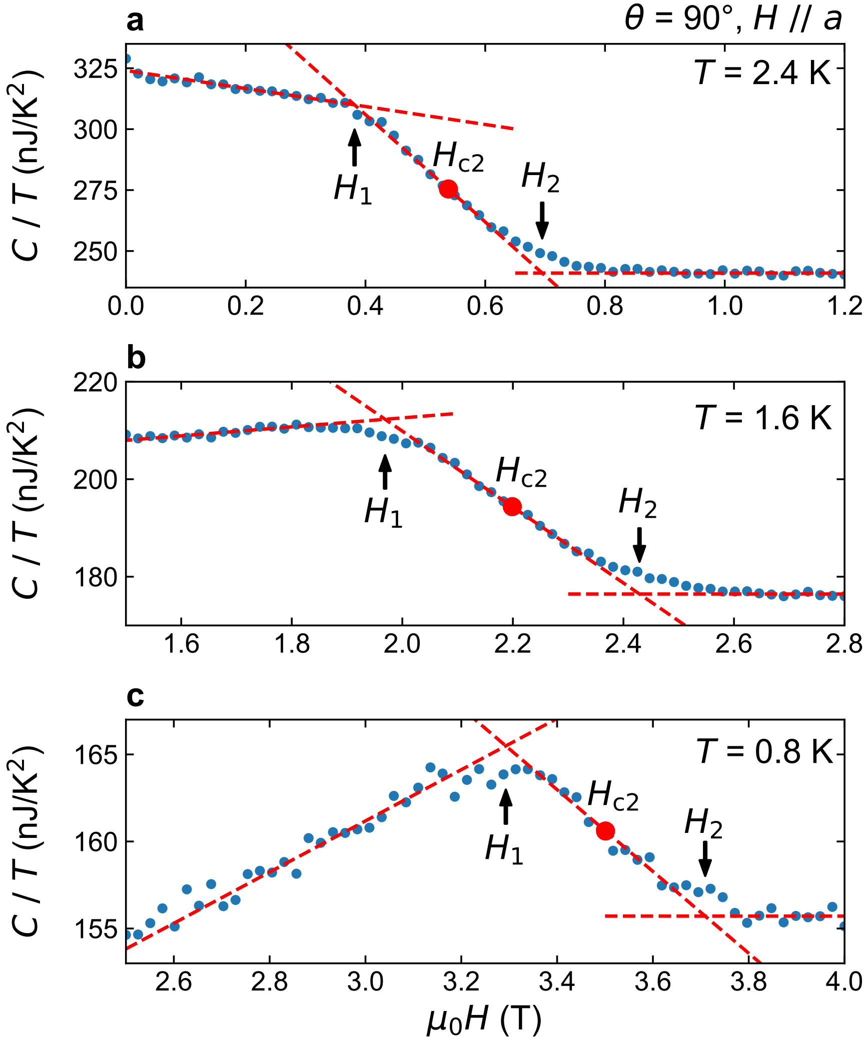

Supplementary Fig. 4 Representative magnetic-field strength dependence of the heat capacity near H_{c2}. a. Magnetic field dependence of the heat capacity at 2.4 K and $\phi = 0°$. The red broken lines are results of linear fittings of the $C(H)/T$ curves to evaluate H_{c2}. The intersections of the fit results are defined as H_1 and H_2 as illustrated in the figure. Then, H_{c2} is defined as $H_{c2} = (H_1 + H_2)/2$. **b** and **c**. Same plots but for 1.6 K and 0.8 K.

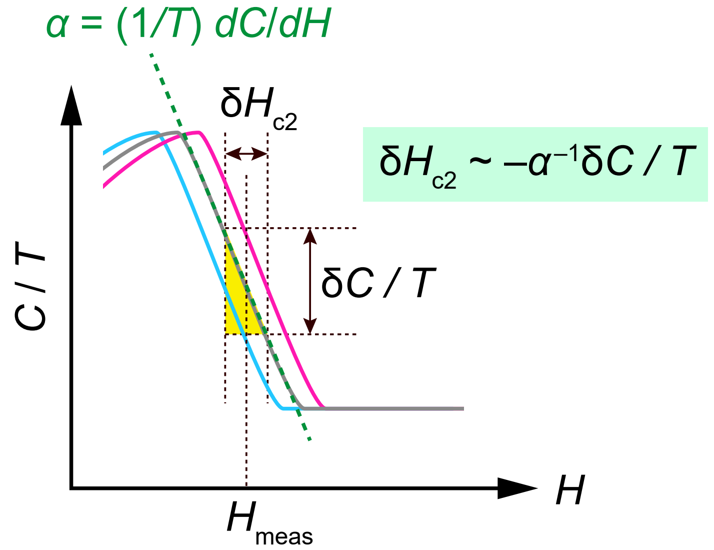

Supplementary Fig. 5 Schematic description of H_{c2} anisotropy evaluation from in-plane specific heat anisotropy. Here, the field strength dependence of $C(H)/T$ has a slope $\alpha = (1/T)dC/dH$ around the upper critical field H_{c2}. When H_{c2} changes by a small value δH_{c2}, the $C(H)/T$ curve exhibits a horizontal shift as depicted by the thick light-blue and pink curves. Thus, if we measure C/T at a fixed field H_{meas} close to the mid-point of the SC transition, the specific heat changes by $\delta C/T$. With simple geographic consideration, δH_{c2} and $\delta C/T$ are related through the formula $(\delta C/T)/(\delta H_{\mathrm{c2}}) \simeq -\alpha$, leading to $\delta H_{\mathrm{c2}} \simeq -\alpha^{-1}\delta C/T$.

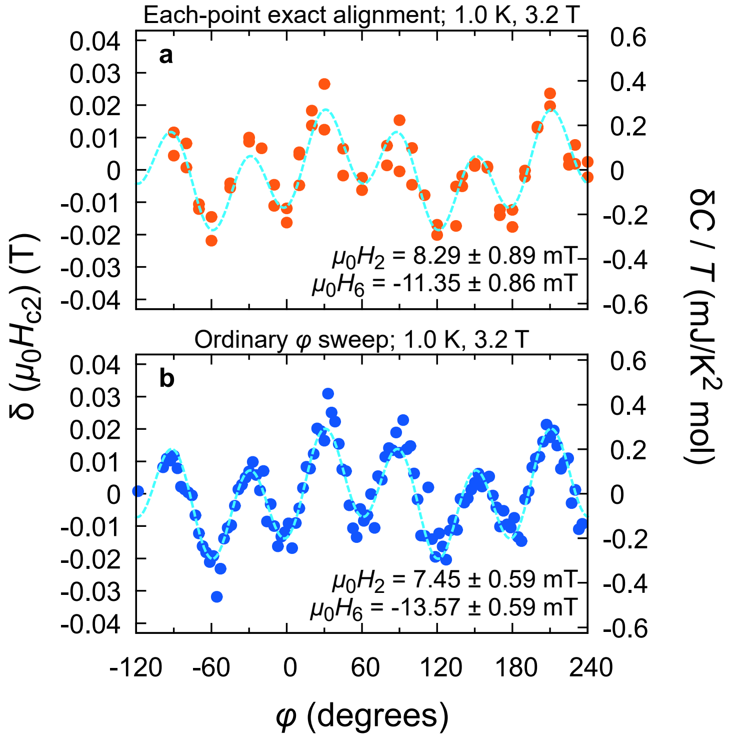

Supplementary Fig. 6 In-plane anisotropy examined under complete absence of field misalignment. **a** Data obtained with the each-point exact alignment method. Here, we performed θ sweep at each ϕ, and then picked up the specific heat at the peak position. This means that accuracy of the field alignment is checked each time after we change ϕ. **b** Data obtained with the ordinary ϕ sweep method. The two data sets, as well as the fitting results shown in the graphs, agree with each other within experimental uncertainties. This manifests that the observed anisotropy is fully intrinsic.

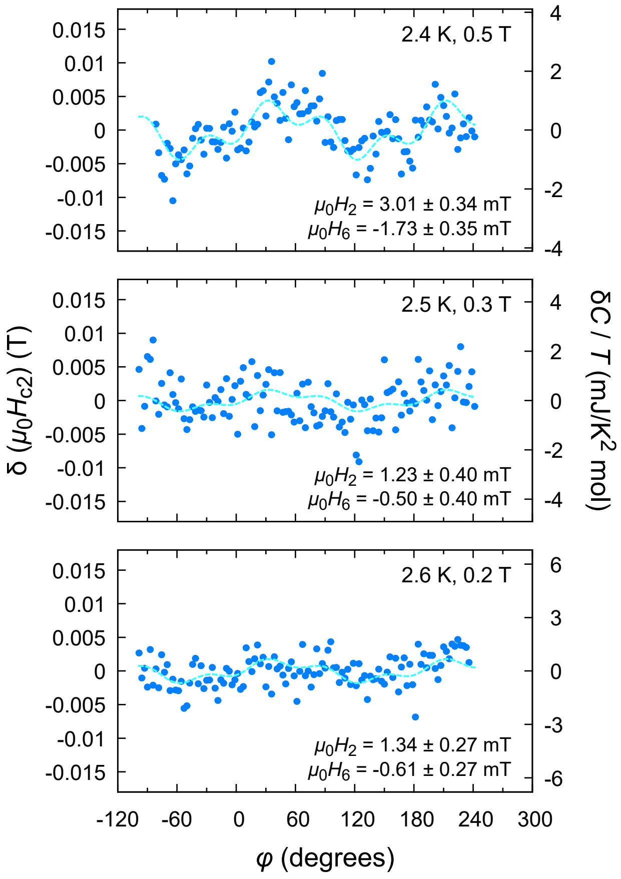

Supplementary Fig. 7 **ΔH_{c2} vs. ϕ curves of CsV_3Sb_5 at 2.4, 2.5 and 2.6 K.** Fitting results using functions with six and two-fold sinusoidal terms are shown with the broken curves. Obtained fitting parameters are written in each panel. The corresponding specific-heat modulation values are shown in the right axes.

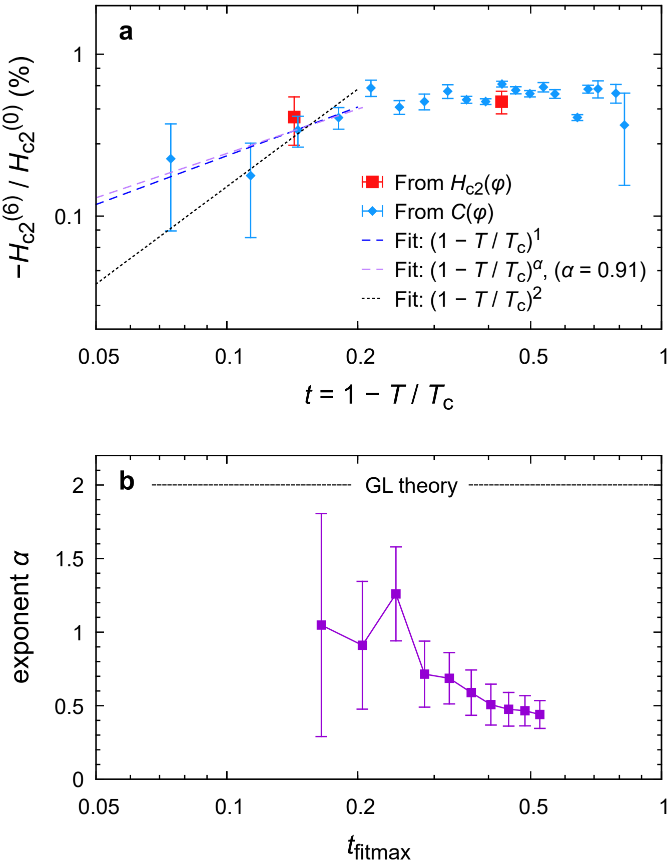

Supplementary Fig. 8 Detailed analysis of the temperature dependence of the six-fold oscillation amplitude near T_c. a. Log-log plot of $-H_{c2}^{(6)}/H_{c2}^{(0)}$ as a function of the reduced temperature $t = 1 - T/T_c$. This graph is intended to investigate the temperature dependence of the hexagonal anisotropy $-H_{c2}^{(6)}/H_{c2}^{(0)}$ above 2.3 K ($1 - T/T_c \lesssim 0.18$), where this ratio exhibits downturn as shown in Fig. 5**a**. The error bar represents asymptotic standard errors of sinusoidal fittings of each $H_{c2}(\phi)$ data set. This log-log plot show that $-H_{c2}^{(6)}/H_{c2}^{(0)}$ is linearly proportional to $1 - T/T_c$ (blue broken line), which is distinct from the quadratic behavior $\propto (1 - T/T_c)^2$ (black dotted line) predicted by standard GL theories. Both curves are obtained by fittings in the range $0.05 < 1 - T/T_c < 0.2$. We also performed fittings with $\propto (1 - T/T_c)^\alpha$ using the exponent α as a fitting parameter. With the fitting range $0.05 < 1 - T/T_c < 0.2$, the fitting yields $\alpha = 0.91$ as shown with the purple broken curve. **b**. Fitting-range dependence of the exponent α. The error bars show asymptotic standard errors of fittings. Here, to avoid ambiguities originating from the choice of fitting range, we performed fittings with $\propto (1 - T/T_c)^\alpha$ by changing the fitting range as $0.05 < 1 - T/T_c < t_{fitmax}$ and the obtained α is plotted against t_{fitmax}. Irrespective of the fitting range, α is substantially less than 2, which is expected for standard GL theories.

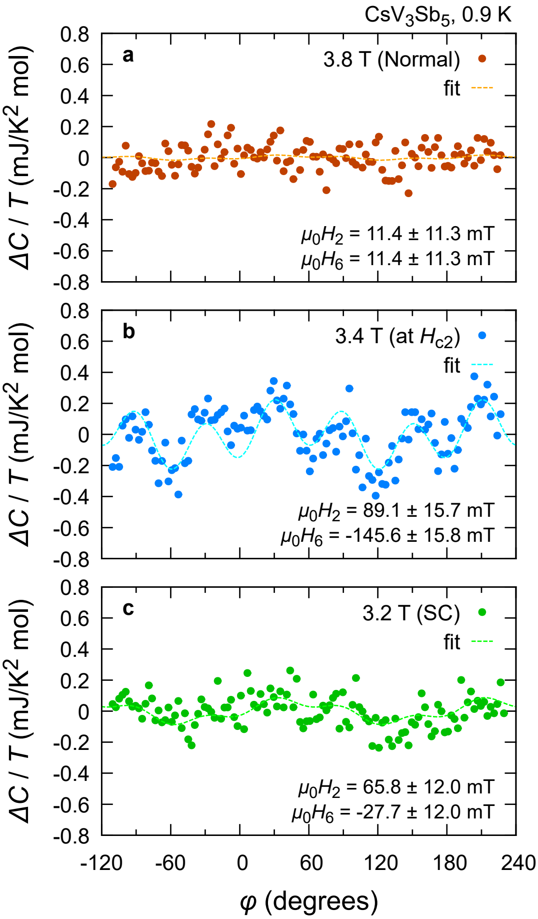

Supplementary Fig. 9 Normal-state in-plane azimuth angle φ dependence of the specific heat of CsV_3Sb_5, compared with those in the SC state. Here, the data in **a** are the specific heat measured at 0.9 K under 3.8 T, which is higher than H_{c2} at this temperature ($\mu_0 H_{c2} = 3.4$ T; see Fig. 2**c**). There is no detectable anisotropy in the normal-conducting CDW state above H_{c2}. For comparison, data measured at H_{c2} and below H_{c2} are shown in panels **b** and **c**, respectively. Results of sinusoidal fittings are shown with broken curves. As written in the graphs, the oscillatory components $H_{c2}^{(2)}$ and $H_{c2}^{(6)}$ exhibit non-monotonic field dependence, peaked at H_{c2}. This non-monotonic behavior exclude possibility that the oscillation originate from certain backgrounds such as thermometer/heater heat capacity or thermometer magnetoresistance.

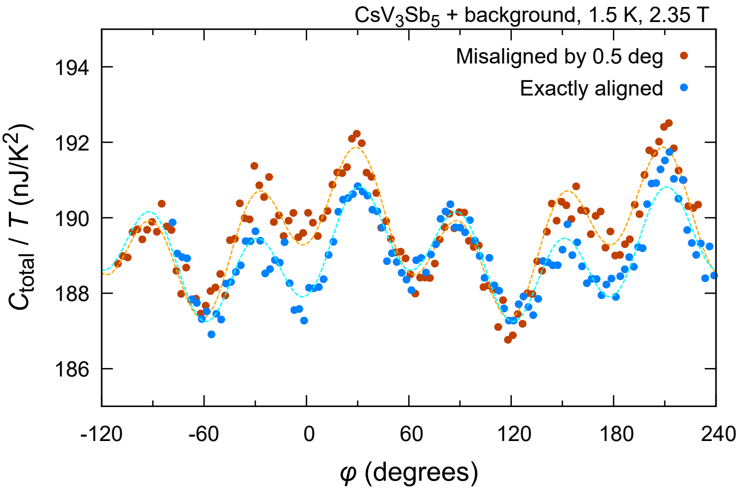

Supplementary Fig. 10 Comparison of raw data measured with and without small field misalignment. We here compare ϕ dependence of the heat capacity with exact alignment and that with slight ($\sim 0.5^{\circ}$) misalignment. The specific heat oscillation is quite sensitive to the misalignment. Such high field-angle sensitivity is not expected for thermometer magnetoresistance effects.

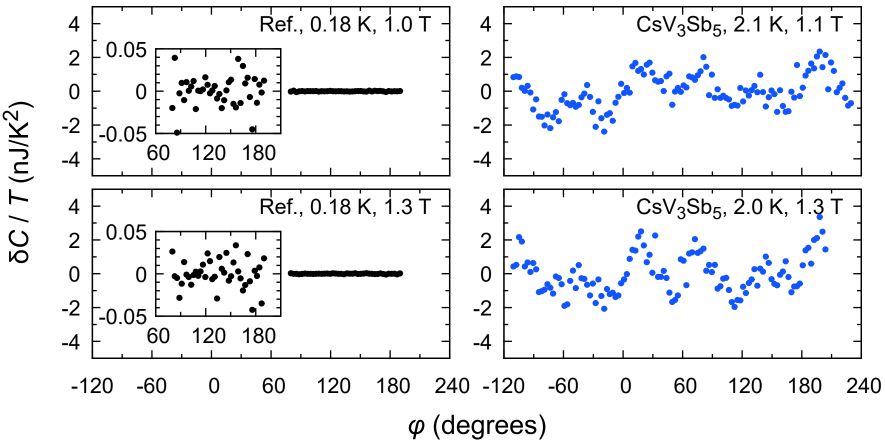

Supplementary Fig. 11 Comparison of field-angle-dependent heat capacity with and without a CsV_3Sb_5 sample. The left figures show data measured with a silver-metal sheet as a reference sample, whereas the right figures show angle-dependent heat capacity with the CsV_3Sb_5 sample. Due to a limited number of available reference-data sets, we here compare data measured with similar magnetic field strength but with different temperatures. Nevertheless, it is expected that extrinsic anisotropies originating from the magnetoresistance of the thermometer and heater should be larger at lower temperatures because magnetoresistance of Ru-oxide thermometer chips are known to be more significant at low temperatures. Also, we should comment that the signal-to-noise ratio is enhanced in the reference data because of large resistance of the thermometer at low temperatures, as well as the inverse relation between heat capacity and measured temperature oscillation ($C \propto 1/T_{\rm ac}$). Therefore, the data in the right panels provides the upper limit of the extrinsic anisotropy due to thermometer magnetoresistance. As shown in the insets, anisotropy in ΔC of the reference data is absent with the resolution of 0.05 nJ/K^2, which is less 2% of the observed oscillation with the sample. Therefore, any spurious anisotropy due to thermometer magnetoresistance is negligible.

Supplementary Note 1

Sugawara *et al.* evaluated the out-of-plane resistance ρ_z under in-plane magnetic field $H \parallel xy$ for a simple quasi-two-dimensional metal with elliptic-cylinder Fermi surface [57]. The magnetoresistance under magnetic field $\boldsymbol{H} = (H\cos\phi, H\sin\phi, 0)$ is given as

$$\rho_z(H,\phi) = \rho_z(H=0)\sqrt{1+\frac{AH^2}{\boldsymbol{v}_{\mathrm{F}}(\phi+\pi/2)^2}}, \tag{1}$$

where A is a field-independent parameter and $\boldsymbol{v}_{\mathrm{F}}(\phi+\pi/2)$ is the in-plane Fermi velocity perpendicular to the magnetic field. Assuming that x and y directions are the principal directions of the nematic $\boldsymbol{v}_{\mathrm{F}}$ anisotropy, we obtain

$$\rho_z^2(H \parallel x) = \rho_z^2(H=0)\left[1+\frac{AH^2}{{v_{\mathrm{F}y}}^2}\right] \tag{2}$$

$$\rho_z^2(H \parallel y) = \rho_z^2(H=0)\left[1+\frac{AH^2}{{v_{\mathrm{F}x}}^2}\right] \tag{3}$$

From this relation, we easily obtain

$$\frac{v_{\mathrm{F}x}}{v_{\mathrm{F}y}} = \sqrt{\frac{\rho_z^2(H \parallel x)-\rho_z^2(H=0)}{\rho_z^2(H \parallel y)-\rho_z^2(H=0)}}. \tag{4}$$

Thus, the Fermi velocity anisotropy $v_{\mathrm{F}x}/v_{\mathrm{F}y}$ can be estimated using experimental data.

Using the c-axis resistivity of $\mathrm{CsV_3Sb_5}$ measured under in-plane magnetic field reported in Ref. [32], we evaluate $\sqrt{[\rho_c^2(H \parallel a)-\rho_c^2(H=0)]/[\rho_c^2(H \parallel a^*)-\rho_c^2(H=0)]}$ under various conditions as tabulated below. The $\boldsymbol{v}_{\mathrm{F}}$ anisotropy evaluated in this way is 9-14% depending on measurement conditions. Notice that this evaluation ignores possible anisotropy

in the scattering time due to the CDW order, and thus may overestimate the Fermi-velocity anisotropy.

Using the GL theory, the upper critical field H_{c2} with in-plane field direction ϕ is given by

$$H_{\mathrm{c2}}(\phi) = \frac{\Phi_0}{2\pi\xi_z\xi(\phi+\pi/2)}, \tag{5}$$

where ξ_z is the out-of-plane GL coherence length and $\xi(\phi+\pi/2)$ is the in-plane GL coherence length perpendicular to the field. The GL coherence length nearly matches the BCS coherence length $\xi_{\mathrm{BCS}} = \hbar v_{\mathrm{F}}/\pi k_{\mathrm{B}} T_{\mathrm{c}}$ at temperatures much lower than T_{c}. Thus, the in-plane H_{c2} anisotropy should satisfy the relation

$$\frac{H_{\mathrm{c2}}(H \parallel a)}{H_{\mathrm{c2}}(H \parallel a^*)} \sim \frac{|\boldsymbol{v}_{\mathrm{F}}^{a}|}{|\boldsymbol{v}_{\mathrm{F}}^{a^*}|}. \tag{6}$$

Notice that, in our experiment, the in-plane two-fold H_{c2} anisotropy $H_{\mathrm{c2}}^{(2)}$ is given by the half of the peak-to-peak anisotropy. Therefore, the 9-14% anisotropy in $\boldsymbol{v}_{\mathrm{F}}$ listed in the table would correspond to the ratio $H_{\mathrm{c2}}^{(2)}/H_{\mathrm{c2}}^{(0)}$ of around 4.5-7%. This anisotropy is much greater than that observed in our experiment (less than 1%). Nevertheless, the magnetoresistance anisotropy has the principle axis along the a or a^* axis, whereas the principle axis of the H_{c2} anisotropy is 45-degree tilted from the a axis. Because of this quantitative difference, it is so far difficult to attribute the nematic H_{c2} anisotropy solely to the possible Fermi velocity anisotropy induced by the CDW order.

Supplementary Table 1 In-plane Fermi-velocity anisotropy of $\mathrm{CsV_3Sb_5}$ evaluated using c-axis transport data taken under in-plane magnetic fields [32].

T	$\mu_0 H$	$\rho_c(H=0)$	$\rho_c(H \parallel a)$	$\rho_c(H \parallel a)$	$\lvert \boldsymbol{v}_{\mathrm{F}}^{a} \rvert / \lvert \boldsymbol{v}_{\mathrm{F}}^{a^*} \rvert$ from Eq. (4)
2 K	4 T	119 $\mu\Omega$cm	241 $\mu\Omega$cm	226 $\mu\Omega$cm	1.09
2 K	6 T	119 $\mu\Omega$cm	322 $\mu\Omega$cm	289 $\mu\Omega$cm	1.14
10 K	5 T	134 $\mu\Omega$cm	292 $\mu\Omega$cm	264 $\mu\Omega$cm	1.14
15 K	5 T	151 $\mu\Omega$cm	300 $\mu\Omega$cm	278 $\mu\Omega$cm	1.11
